# Integrated Care in Epilepsy Management: A Scoping Review of the Models and Components of Health and Social Care Delivery

**DOI:** 10.5334/ijic.7659

**Published:** 2024-03-08

**Authors:** Samantha Spanos, Karen Hutchinson, Tayhla Ryder, Frances Rapport, Nicholas Goodwin, Yvonne Zurynski

**Affiliations:** 1 Centre for Healthcare Resilience and Implementation Science, Australian Institute of Health Innovation, Macquarie University, Sydney, Australia; 2 Central Coast Local Health District, Gosford, NSW, Australia; 3 CanTeen Australia, Sydney, Australia; 4 Central Coast Research Institute for Integrated Care, University of Newcastle, Gosford, NSW, Australia; 5 NHMRC Partnership Centre for Health System Sustainability, Australian Institute of Health Innovation, Macquarie University, Sydney, Australia

**Keywords:** epilepsy, integrated care, shared care, person-centred care

## Abstract

**Introduction:**

Epilepsy is the most common neurological condition globally. Integrating health and social care is fundamental in epilepsy management, but the scope of progress in this area is unclear. This scoping review aimed to capture the range and type of integrated care components and models in epilepsy management.

**Methods:**

Four databases were searched for articles published since 2010 that reported on integrated care in epilepsy. Data were extracted and synthesised into components of integrated care that had been implemented or recommended only. Models of integrated care were identified, and their components tabulated.

**Results:**

Fifteen common and interrelated components of integrated care emerged that were aligned with four broad areas: healthcare staff and pathways (e.g., epilepsy nurses); tasks and services (e.g., care coordination); education and engagement (e.g., shared decision making); and technology for diagnosis and communication (e.g., telehealth). Twelve models of integrated care were identified; seven were implemented and five were recommended.

**Discussion:**

There is a growing evidence-base supporting integrated, person-centred epilepsy care, but implementation is challenged by entrenched silos, underdeveloped pathways for care, and deficits in epilepsy education.

**Conclusion:**

Integrating epilepsy care relies on changes to workforce development and policy frameworks to support whole-of-system vision for improving care.

## Introduction

Epilepsy is the most common neurological condition that affects 50 million people worldwide, with an estimated 5 million people diagnosed each year [1]. Epilepsy is a condition that does not discriminate; it can affect individuals of all ages, genders, and socioeconomic backgrounds [2,3,4]. People living with epilepsy (PLWE) experience physical, psychological, and psychosocial impacts and require healthcare from interdisciplinary teams of clinicians as well as psychological and social care [5,6,7]. For these reasons, the National Institute for Health and Care Excellence recommends integrated care, care coordination and involvement of multidisciplinary teams (MDTs) to support all stages of care required by PLWE over their life course [8]. However, little is known to what extent these guidelines have been implemented in practice and which components of integrated care models have been adopted.

Approximately two-thirds of PLWE can achieve ‘seizure freedom’ with anti-seizure medication [1]. The remaining one third, however, live with more complex and difficult-to-treat epilepsy (often known as refractory or drug-resistant epilepsy) that is not effectively managed by anti-seizure medication alone, and for whom brain surgery is a potential treatment option [2]. However, anti-seizure medication can contribute to significant physical, psychological, and cognitive adverse effects, and thus even when an individual achieves ‘seizure-freedom’, epilepsy management can still significantly impact their lives [4].

Epilepsy has many different aetiologies, and is often associated with significant comorbidities and disability [3]. Appreciating the greater prevalence of epilepsy in individuals who have an intellectual disability, cerebral palsy, brain tumours or brain trauma [2] is important for timely diagnosis, prognosis, and quality of life [4]. In addition, the greater psychological distress and higher rates of mental illness experienced by PLWE than those without the condition [4] can contribute to relationship and family difficulties, employment issues, and lower educational attainment [9].

Considering the heterogeneity and complexity of managing epilepsy, PLWE often require ongoing health and social care throughout their lives [7]. Yet the challenge of epilepsy management for healthcare professionals (HCPs), especially in primary care settings, who may have limited knowledge and expertise about epilepsy, can significantly influence the care experience for PLWE and their families [6]. Childhood epilepsy can be associated with other conditions such as tuberous sclerosis complex, a genetic disorder with highly heterogeneous signs and symptoms, which can further complicate epilepsy diagnosis and management [10]. It is internationally recognised that epilepsy-related health and social care systems lack standardisation and formalised clinical pathways [6,7]. Poor communication across service providers and ambiguity in roles and responsibilities across the care continuum has resulted in entrenched fragmentation of epilepsy care services [5], which can severely impact the timing of referrals, access to targeted treatments, and the efficiency of care delivery for PLWE [2,6].

Integrating care between different epilepsy services and across sectors can support a holistic, person-centred approach, where health care and other services are more efficiently and effectively coordinated around the needs of PLWE, similar to others living with chronic and complex conditions [11]. Integrated care is seen as a means of promoting value-based healthcare, which advocates centring health outcomes that matter to PLWE and their family, improving the quality and cost effectiveness of care, and improving the experience of service providers [12,13].

Applying integrated person-centred care to epilepsy management could provide a more equitable, cost-effective, high quality care solution to meet the specific health and wellbeing needs of PLWE. A multitude of diverse approaches to integrated person-centred care exist across health and social care for epilepsy and other chronic conditions, but there is limited understanding of the components of integrated care models and how they have been combined for epilepsy management.

This scoping review aims to better understand the literature on integrated care in epilepsy management. It seeks to provide new knowledge to the broader literature on integrated care since it goes beyond a descriptive understanding of the necessary components of integrated care, to understand how these components have been implemented in current models, with recommendations for including components that models seemingly omit. This review consolidates and extends the findings of Hutchinson et al.’s 2020 working paper [14], guided by two key questions:

1. What are the common components of integrated care approaches that have been recommended or implemented in epilepsy management?
2. What models of integrated care have been recommended or implemented in epilepsy management?

## Research Methods

A scoping review of the peer reviewed literature was conducted in accordance with the Preferred Reporting Items for Systematic Reviews and Meta-Analyses Extension for Scoping Reviews (PRISMA-ScR) guidelines [15].

### Search strategy and identification of studies

A comprehensive search strategy (see Appendix 1) was developed encompassing commonly used synonyms and terms relating to integrated care (e.g., shared care) [11]. Any models, interventions, and practices with the objective of addressing fragmentation in care delivery were included within the scope of integrated care or person-centred care.

Three literature searches were conducted. The initial search of two databases, PubMed and Web of Science, was conducted from January 2010 to August 2020 to focus on contemporary integrated care perspectives and approaches. The search was updated in May 2022, covering the period August 2020 to May 2022. In addition, to increase comprehensiveness of the review, the search was undertaken in additional databases (Medline and Embase) covering January 2010 to May 2022. To improve the currency of the search, another updated search was conducted of all four databases covering the period May 2022 to November 2022.

### Study selection

All database search results (January 2010 to November 2022) were combined and uploaded into Endnote where duplicate records were identified and removed. Titles and abstracts of articles were screened by six reviewers (KH, TR, CP, SS, YZ, FR) to assess compliance with inclusion and exclusion criteria (Table 1). To improve inter-rater reliability, 25% of the abstracts were screened by at least two reviewers. Any disagreements or uncertainties were discussed by the team until consensus was reached. Once consensus was reached based on title and abstract, full-text review and data extraction was carried out by six reviewers (KH, TR, CP, SS, YZ, FR).

**Table 1:** Inclusion and exclusion criteria for study selection in scoping review.


Inclusion criteria:

Articles reporting on epilepsy as a key focus, although may be applicable to other long-term and complex conditions Articles articulating a definition or description of the concept or principles of integrated care (and related terms like coordinated care) Articles describing a model of care, or components of an integrated approach to improve the organisation and delivery of care in epilepsy, that has been put into practice or is being recommended

**Exclusion criteria:**

Articles reporting on biomedical or clinical studies, or articles that are meta-analyses Articles published prior to 2010 Editorials, commentaries, letters, conference abstracts Grey literature No full-text available Languages other than English


### Data charting and synthesis

Data were extracted into a purpose-designed Excel spreadsheet and included general study characteristics (e.g., publication year, country, methodology, population), models of integrated care for epilepsy management, whether the models had been implemented or only recommended and not yet implemented, and specific components within each model. Where articles did not specify a model of care, but described components of integrated care in epilepsy, data on these components were extracted. Data in literature reviews (including reviews conducted as part of an empirical study) were extracted as presented in the article; we did not search for any additional information about the models or components that were described in these reviews. Data on integrated care components were organised and synthesised into common categories (or areas) and were quantified and tabulated to analyse the frequency of each component across the reviewed articles and across various models of care. Data on models of care were quantified to determine the frequency of implemented or recommended-only models.

Given that the purpose of this review was to scope the literature on integrated epilepsy care rather than to evaluate the effectiveness of particular interventions, a methodological quality assessment was not conducted [15].

## Results

### Search results

**Figure 1:**
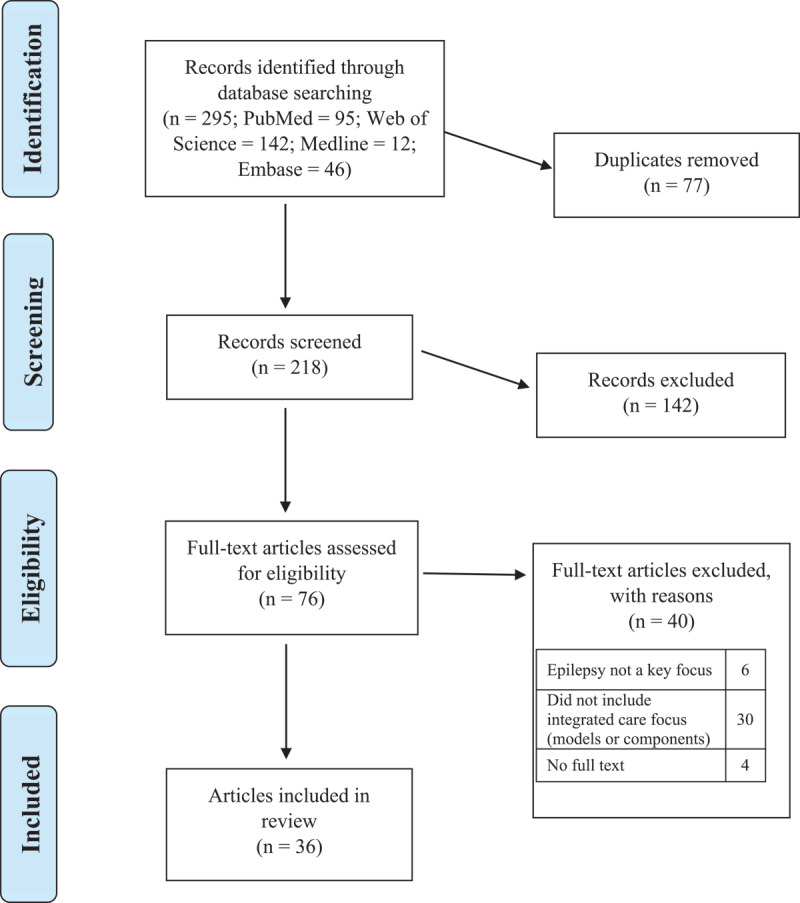
PRISMA flowchart displaying the process of identification and selection of included articles.

The study selection process is summarised in the PRISMA-ScR flow chart (Figure 1). A total of 295 articles were identified. Following the removal of duplicate studies (*n* = 77), a total of 218 articles were screened by title and abstract, and 142 were excluded because they did not meet criteria, leaving 76 articles eligible for full-text review. Forty were subsequently excluded for failing to meet the eligibility criteria, leaving 36 articles included for data extraction and analysis.

### Characteristics of included articles

The characteristics of included articles are displayed in Table 2 and the full list of included articles is provided in Appendix 2.

**Table 2:** Frequency of study characteristics included in scoping review.


STUDY CHARACTERISTICS (*N* = 36)	*n* (%)

***Publication year***	

2010–2013	6 (17)

2014–2017	5 (14)

2018–2022	25 (69)

***Study location***	

Europe	21 (58)

North America	11 (31)

Australia	3 (8)

South America	1 (3)

Article type or study design*	

Descriptive	16 (44)

Literature review	8 (22)

Retrospective cohort	7 (19)

Prospective cohort	2 (6)

Narrative review	2 (6)

Synthesis of expert recommendations	1 (3)

Delphi	1 (3)

Case study	1 (3)

Case series	1 (3)

***Implementation or recommendation focus***	

Refers to implemented model of care	10 (28)

Refers to implemented components, but not full models, of care	9 (25)

Recommends new model of care that is not implemented	5 (14)

Recommends new components, but not full models, of care, that are not implemented	12 (33)

***Components of integrated care***	

*Healthcare staff & pathways*	

Multidisciplinary teams	22 (61)

Cross-sector collaboration	19 (53)

CPGs or care pathways	13 (36)

Epilepsy nurses	7 (19)

*Tasks & services*	

Psychosocial services	19 (53)

Care coordination	13 (36)

Care management plans	11 (31)

Transition services	5 (14)

Surgical evaluation	3 (8)

*Education & engagemen*t	

Shared decision making	19 (53)

Education for PLWE and families	15 (42)

Education for HCPs	13 (36)

*Technology for diagnosis & communication*	

Shared electronic medical records	12 (33)

Telehealth	8 (22)

Digital health tools	5 (14)


*N*, total number of articles included in scoping review; *n*, number of articles included in the frequency analysis; CPGs, clinical practice guidelines; PLWE, people living with epilepsy; HCPs, healthcare professionals. * Three articles utilised more than one type of study design.

### Integrated care components and models

Fifteen interrelated components were identified in the current review to be important for integrated epilepsy management. Components reported on were either in the context of a model of care (*n* = 15), or as standalone components (*n =* 21). From the 15 articles that reported on models of care, 12 full models were identified (seven implemented and five recommended but not implemented) (see Tables 3 and 4).

**Table 3:** Implemented models of integrated care in epilepsy management.


IMPLEMENTED MODEL	COMPONENTS*

National Clinical Programme for Epilepsy [42,46,47,48,49,50]	*Cross-sector collaboration*: community supports, secondary care, tertiary care *MDTs*: nursing, primary care, neurology, epileptology, emergency medicine *CPGs & care pathways*: Integrated care pathway for emergency department seizure management *ENs*: triage and connect PLWE with services across sectors *Care coordination*: often performed by nurse or EN *CMPs*: ENs play a key role in assessing care arrangements *Education for HCPs*: education on care pathways for new and junior staff *Education for PLWE & families*: education sessions provided by HCPs *SDM*: facilitated by EN and through CMPs *Telehealth*: telephone advice line virtual clinics to enhance communication between HCPs *Shared EMRs:* shared across epilepsy services to improve care coordination

Epilepsy nurse-led model [42]	*Cross-sector collaboration*: primary, secondary, tertiary care sectors *MDTs*: doctors, specialists, nurses, ENs *Care coordination*: ENs coordinate care across health and social care services *Psychological services*: ENs provide psychological and wellbeing support *CMPs*: ENs complete comprehensive treatment assessments *Education for PLWE & families*: ENs provide person-centred education to promote confidence to self-manage *SDM*: ENs engage in SDM with PLWE and families to enhance care outcomes *Telehealth:* ENs operate telephone advice lines to assist with PLWE and family concerns *Shared EMRs:* utilised by ENs as a guide to structure healthcare information

Integrated care pathway for seizure management in emergency department [46,47]	*MDTs*: inter-specialty approach; emergency department hospital staff and rapid clinic staff *CPGs & care pathways*: embedded national guidelines into care pathway *Education for HCPs*: provided for junior doctors and new staff *ENs*: EN service for triage and discharge follow-up

Integrated care pathway for homeless PLWE [41]	*Cross-sector collaboration*: health, community sectors *MDTs*: community services staff, hospital staff, epilepsy specialist *CPGs & care pathways:* new care pathway for homeless PLWE *CMPs*: jointly created treatment plans disseminated across providers *Shared EMRs*: care plans and outcomes shared on hospital records, national epilepsy patient record, and community records

Urgent epilepsy clinic [43]	*MDTs*: epilepsy physicians, triage nurses, social worker *Psychosocial services*: clinic focused on education, counselling, and addressing psychosocial risk factors *CMPs*: seizure action plans deployed to improve knowledge around home seizure management *Education for HCPs*: epilepsy education on seizure management *Education for PLWE & families*: facilitated by seizure action plan development *Shared EMRs*: used to manage the proper dosing of emergency seizure medications

Neurocare service [44]	*Cross-sector collaboration*: primary care, hospital, community sectors *MDTs*: hospital discharge team and community neurological nurses *Care coordination*: community neurological nurses helped PLWE navigate multiple providers *CMPs*: community neurological nurse-led individual goal setting and action planning *Education for PLWE & families*: community neurological nurses provided education about medications, symptom management and lifestyle changes *SDM*: community neurological nurses worked with PLWE on their self-management planning *Telehealt*h: care delivery modes included telephone, videoconferencing, email, and text messaging

Children and Young People’s Health Partnership Evelina London Model [45]	*Cross-sector collaboration*: primary care, hospital, community sectors *MDT*s: children’s nurses, general practitioners, paediatricians, mental health specialists *Care coordination*: children’s nurses coordinate and deliver care across sectors *Psychosocial services*: biopsychosocial pre-assessment to inform early intervention care *SDM*: active family involvement in decision making processes and shared learning *Telehealth*: nurses communicated with families via telephone, email, and text messaging


*As reported within relevant articles; MDT, multidisciplinary team; PLWE, people living with epilepsy; HCP, healthcare professional; EN, epilepsy nurse; CPGs, clinical practice guidelines; CMPs, care management plans; SDM, shared decision-making; EMR, electronic medical record.

**Table 4:** Models of integrated care in epilepsy management that were recommended but not implemented.


RECOMMENDED MODEL	COMPONENTS*

Hub-and-spoke [36]	*Cross-sector collaboration*: community, health sectors *MDTs*: genetics, neurology, nephrology, psychology, psychiatry, paediatrics *Care coordination*: dedicated tuberous sclerosis complex specialist coordinator *Psychosocial services*: provision of supportive care including genetic counselling *Transition services*: collaboration between paediatric and adult clinics *CMPs*: tailored to support management and surveillance of symptoms *Education for HCPs*: provide a structure to facilitate education of HCPs *Education for PLWE & families*: individualized education plans *SDM*: high level of involvement by PLWE and families

Integrated care for children and young people [37]	*Cross-sector collaboration:* health, education, social care, voluntary sectors *MDTs*: nursing, primary care, paediatrics, neurology, allied health, mental health *CMPs*: individualised and developed by MDTs *Education for HCPs*: education and training courses for providers *SDM*: working in partnership with children, young people, and families *Shared EMRs*: co-produced formal tools to enhance communication; key feature is a national epilepsy registry with up-to-date data to inform care *Digital health tools*: utilised by specialists to maximize diagnostic accuracy

Chronic disease management [38]	*Cross-sector collaboration*: health, social care sectors *MDTs*: primary care HCPs, ENs, epilepsy specialists *CPGs & care pathways*: embed CPGs that support clinical decisions *ENs*: integral part of epilepsy care coordination and education provision *Care coordination*: EN coordinates and monitors care arrangements *Education for HCPs*: education support, incentives, and CPGs are particularly important for primary care clinicians *Education for PLWE & families*: education and self-management are key to managing epilepsy *SDM*: promotes continuity through shared-care partnerships *Shared EMRs*: to standardise clinical information and educate staff

Paediatric acute seizure care pathway [39]	*Cross sector collaboration*: health, community, education sectors *MDTs*: emergency department staff, hospital physician, neurologist, epileptologist *CPGs & care pathways*: identifying where CPGs are not integrated into practice and providing recommendations *Care coordination*: nurse navigator or designated care coordinator *Psychosocial services*: psychosocial counselling for carers *CMPs*: preventative seizure action plan in prehospitalisation settings *Education for HCPs*: on seizure action plans and rescue medication *Education for PLWE & families*: seizure action plans facilitate education on seizure management *Shared EMRs*: for sharing seizure and medication data and supporting monitoring and coordination of care for PLWE *Digital health tools*: advanced seizure detection technology (e.g., electroencephalogram)

Model of transition (paediatric to adult) [40]	*MTDs*: single combined clinic for child and adult services from paediatrics, neurology, psychology and ENs *CPGs & care pathways*: guideline analysis to inform transition services *Psychosocial services*: focused on psychosocial and communication needs of young people *Transition services*: knowledge exchange and information needs during transition from children’s to adult services *Education for PLWE & families*: age- and language-appropriate written information on safety and seizure management *SDM*: to understand communication needs of PLWE and families


*As reported within relevant articles; HCP, healthcare professional; MDT, multidisciplinary team; PLWE, people living with epilepsy; EN, epilepsy nurse; CPGs, clinical practice guidelines; CMPs, care management plans; SDM, shared decision-making; EMR, electronic medical record.

Of the 15 components identified, 12 were included in an implemented model, two were proposed in a recommended model, and one was not included in any model of care but was recommended as a standalone component. Table 5 displays the 15 components, the seven implemented models, and five recommended models, indicating the inclusion status of each component within the models of care.

**Table 5:** Integrated care components across implemented and recommended models.


	HEALTHCARE STAFF & PATHWAYS				TASKS & SERVICES					EDUCATION & ENGAGEMENT			TECHNOLOGY FOR DIAGNOSIS & COMMUNICATION		
			
	MDT	CROSS SECTOR COLLABORATION	CPG & CARE PATHWAYS	ENS	PSYCHOLOGICALSERVICES	CARE COORDINATION	CMPS	TRANSITION SERVICE	SURGICAL EVALUATION & SUPPORT*	SDM	EDUCATION FOR HCPS	EDUCATION FOR PLWE & FAMILIES	SHARED EMRS	TELEHEALTH	DIGITAL HEALTH TOOLS

**IMPLEMENTED**															

% models including relevant component	100%	71%	43%	29%	43%	57%	71%	0%	0%	57%	43%	57%	57%	57%	0%

National Clinical Programme for Epilepsy [42,46,47,48,49,50]	Y	Y	Y	Y	N	Y	Y	N	N	Y	Y	Y	Y	Y	N

Epilepsy nurse-led model [42]	Y	Y	N	N	Y	Y	Y	N	N	Y	N	Y	Y	Y	N

Integrated care pathway for seizure management in emergency department [46,47]	Y	N	Y	Y	N	N	N	N	N	N	Y	N	N	N	N

Integrated care pathway for homeless PLWE [41]	Y	Y	Y	N	N	N	Y	N	N	N	N	N	Y	N	N

Urgent epilepsy clinic [43]	Y	N	N	N	Y	N	Y	N	N	N	Y	Y	Y	N	N

Neurocare service [44]	Y	Y	N	N	N	Y	Y	N	N	Y	N	Y	N	Y	N

Children and Young People’s Health Partnership Evelina London Model [45]	Y	Y	N	N	Y	Y	N	N	N	Y	N	N	N	Y	N

**RECOMMENDED**															

% models including relevant component	100%	80%	60%	20%	60%	60%	60%	40%	0%	80%	80%	80%	60%	0%	40%

Hub and spoke [36]	Y	Y	N	N	Y	Y	Y	Y	N	Y	Y	Y	N	N	N

Integrated care for children and young people [37]	Y	Y	N	N	N	N	Y	N	N	Y	Y	N	Y	N	Y

Chronic disease management [38]	Y	Y	Y	Y	N	Y	N	N	N	Y	Y	Y	Y	N	N

Paediatric acute seizure care pathway [39]	Y	Y	Y	N	Y	Y	Y	N	N	N	Y	Y	Y	N	Y

Model of transition (paediatric to adult) [40]	Y	N	Y	N	Y	N	N	Y	N	Y		Y	N	N	N


Y = Yes, N = No, MDT = multidisciplinary team, CPG = clinical practice guidelines, ENs = Epilepsy nurses, CMPs = care management plans, SDM = shared decision making, HCPs = healthcare professionals, PLWE = people living with epilepsy, EMRs = electronic medical records. *Recommended component of integrated care but currently a stand-alone service and not included within a specific model of care.

The 15 components were aligned, across all included articles, with four broad areas: *healthcare staff and pathways, tasks and services, education and engagement*, and *technology for diagnosis and communication*. Nine articles referred to one or more integrated care components that had been *implemented* [16,17,18,19,20,21,22,23,24], and 12 articles *recommended* one or more integrated care components [6,25,26,27,28,29,30,31,32,33,34,35].

#### Healthcare staff and pathways

*Multidisciplinary teams (MDTs):* Twenty-two articles (61%) assessed the role and importance of MDTs in maximising the effectiveness and continuity of care for PLWE, but there was no consistency on the HCPs included in the MDT. Most of these articles (*n* = 13) recommended approaches to MDT organisation to foster optimal care for PLWE across various care settings [6,25,26,28,30,33,34,35,36,37,38,39,40]. The remaining articles reported on the role of MDTs within other components of integrated care (e.g., care coordination) or models of integrated care that have been implemented [22,23,41,42,43,44,45,46,47].

*Cross-sector collaboration:* Nineteen articles (53%) focused on the importance of cross-sector collaboration in integrated care for epilepsy management [6,18,21,22,23,25,30,34,36,37,38,39,42,44,45,48,49,50]. This included collaboration across primary, secondary, and tertiary health sectors [6,34,42], the community sector – including support groups and organisations for PLWE and families [22,36,37], mental health services [23,36], and community HCPs [23,36] – the social care sector [23,30,37,41], the voluntary sector [18,22,25], and the education sector [18,23,25,30,37,39,45].

*Clinical practice guidelines & care pathways:* Thirteen articles (36%) assessed the use of clinical practice guidelines (CPGs) and/or care pathways in epilepsy services. Six articles evaluated care pathways for emergency seizure management for adults [43,46,47] and children [39], for homeless PLWE [41], and for chronic disease management [38]. Seven articles evaluated the use of CPGs: during transition services [19], for the referral of complex epilepsy [6,31], for the treatment of paediatric seizures [18], in telepharmacist medication recommendations [22], in primary care follow-up and management of PLWE [6,29], and in person-and-family centred treatment planning [16].

*Epilepsy nurse services*: Seven articles (19%) focused specifically on the role and importance of epilepsy nurse (EN) services. While three studies recommended opportunities for ENs within integrated care [34,38,50], three studies evaluated existing EN roles within implemented models [42,46,47], and one study evaluated a person-centred nursing communication tool [17].

#### Tasks and services

*Psychosocial services:* Nineteen articles (53%) highlighted psychosocial services in epilepsy care. Most of these articles (*n* = 14) discussed psychosocial services within the MDT context to meet the complex needs of PLWE [22,25,26,27,31,33,35,36,39,40,43,45,49,50]. Three articles proposed new frameworks of care that placed a greater focus on wellbeing and psychosocial care [28,30,32], and two articles emphasised the need to partner with PLWE and families to enhance self-management and emotional support [23,42].

*Care coordination:* Thirteen articles (36%) assessed care coordination, the management and organisation of individual medical needs across their lifetime, in epilepsy care services. Five articles focused specifically on nurse-led care coordination [38,39,42,44,45]. The remaining eight articles focused on the care coordinator role in MDTs [28,33,35,36,48], in identifying the varied and often complex needs of PLWE [6,19], and in assisting with pathways to surgery evaluation [28,31].

*Care management plans:* Eleven articles (31%) discussed care management plans (CMPs) as an important component of integrated epilepsy services. Five of these articles assessed CMPs specific to seizure management [16,18,19,39,43]. Four articles focused on the role of MDTs in creating CMPs [35,36,37,41], and two articles focused specifically on the ENs role [42,44]. One article explored person-and-family centredness in care planning [16].

*Transition from paediatric to adult services:* Five articles (14%) examined transition from paediatric to adult services. Three articles emphasised the role of MDTs in facilitating care transitions [33,35,36]. Two studies focused on transition service gaps, including the appropriate assessment of communication and information needs of young PLWE and their families [40], and the need for sexual and reproductive health counselling for PLWE of childbearing age [27].

*Surgical evaluation and support:* Three articles (8%) focused on surgical evaluation and support for PLWE. These articles assessed challenges in identifying people with refractory epilepsy and barriers to surgical evaluation [6], educational interventions to promote utilisation of epilepsy surgery [31], and palliative care approaches to addressing surgery expectations, goals, and concerns [28].

#### Education and engagement

*Shared decision making:* Nineteen articles (53%) highlighted the need for shared decision making (SDM) between HCPs and families affected by epilepsy. Twelve articles centred on specific strategies that facilitate SDM: forming collaborative partnerships with PLWE and families [23,26,37,38,45] and support organisations [35,36], goal setting and care planning [16,28,42], and creating communication tools for HCPs and PLWE [17,21]. Seven articles discussed the role of strong communication and knowledge exchange with PLWE and families [6,19,25,33,40,44,49].

*Education for PLWE and families:* Fifteen articles (42%) discussed education provision for PLWE and families in epilepsy care services. Articles assessed education around seizure management and medication safety [18,19,26,39,40,43,44], as well as education around self-management and self-care [23,27,31,35,36,38,42,50].

*Education for HCPs:* Thirteen articles (36%) assessed education for HCPs delivering epilepsy services. Most of this research (*n* = 8) examined education for HCPs as a component of a model of care [24,34,35,36,38,39,43,47]. Five articles advocated education for specific HCP skillsets, such as those pertaining to ENs [17], neurologists [28], and referring providers such as general practitioners [6,29,31]. Several articles emphasised the need for education in the primary care sector to support the shift from hospital to primary and community-based care [6,29,34,38].

#### Technology for diagnosis and communication

*Shared electronic medical records*: Twelve articles (33%) explored the use of electronic medical records (EMRs) in integrated epilepsy services. Most of these articles (*n* = 9) assessed the use of EMRs to facilitate information exchange across sectors and service providers involved in epilepsy management [22,34,37,38,39,41,43,45,48]. Two articles emphasised the importance of shared EMRs for ENs [42] and care coordinators [19], and one article examined the perspectives of PLWE with regards to shared EMR use [49].

*Telehealth services:* Eight articles (22%) evaluated telehealth services that had been implemented. Telehealth methods included telephone consults and telephone advice line virtual clinics [19,42,48], store-and-forward teleneurology [24], telepharmacist medication review [22], videoconferencing for information exchange between MDTs, PLWE, and families [23], and email and text messaging care delivery modes [44,45].

*Digital health tools:* Five articles (14%) discussed digital health tools that support HCPs, PLWE, and families in epilepsy management. Four articles highlighted that HCPs should utilise innovative technologies such as clinical decision aids that maximise the accuracy and efficiency of care for PLWE [31,33,37,39]. Four articles assessed tools available to support PLWE and families with education and self-management [20,31,33,39].

## Discussion

### Characteristics of articles

This scoping review identified 36 articles examining integrated care components or models for epilepsy management. Most of these articles were descriptive studies that explored the challenges and opportunities in current integrated epilepsy care practices [6,50]. Literature reviews were also common, and most recommended new integrated care components or models [28,38]. Articles were published across 10 different countries, mostly in the last five years, attesting to the rapidly evolving nature of integrated care [51].

### Recommended and implemented models of integrated care

The twelve models of integrated care identified were comprised of various combinations of integrated care components. No single model, implemented or recommended, captured all of the 15 components identified to be important for epilepsy management. Of the implemented models, the National Clinical Programme for Epilepsy [48] was the most comprehensive since it captured 11 of the 15 integrated care components. Of the models that were recommended but not implemented, the paediatric acute seizure care pathway incorporated the most integrated care components, closely followed by the ‘hub and spoke’ and chronic disease management models.

There are clear gaps between the identified components of integrated care and the actual design and implementation of integrated care models. First, although transition services have been recommended, this component has yet to be implemented within any model of care. Specialised transition services are needed to support young people’s empowerment and prevent disengagement from care, and these include sexual and reproductive health counselling and gender-specific information provision [27,40]. Second, digital health tools have been recommended for inclusion in models of care to support HCPs and PLWE but are yet to be implemented. Suggested approaches for incorporating digital tools into models of care should be leveraged and evaluated [37,39]. Additionally, approaches taken to implement both transition services and digital health tools into models of care for other diagnostic groups can serve as a blueprint from which to build upon and adapt services to the specific needs of PLWE [36,40].

Third, surgical evaluation and support is lacking in both implemented and recommended models of integrated care, and has only been recommended as a standalone service, which might be perpetuating the lack of knowledge about epilepsy surgery and low uptake [31]. Processes for identifying and referring people with complex epilepsies for surgical evaluation are key to ensuring that PLWE are aware of their treatment options [6]. Decision tools and CPGs that support HCPs in diagnosis and referral pathways, enabling more timely surgical evaluations and workup, should be incorporated within models of care and piloted for effectiveness [28,31].

### Components of integrated care and barriers and enablers to their implementation

All of the care models identified in this review, both implemented and recommended-only, included MDTs as a component of integrated care, and nine of the 12 models emphasised cross-sector collaboration as a core component. A predominant focus of the literature was identifying optimal MDT arrangements that improve opportunities for shared, holistic and person-centred care through communication within and across sectors [36,37]. Although ENs were emphasised as central to facilitating person-and-family-centred care [23,43], they were underutilised in both implemented and recommended models of care. The role scope of ENs both within MDTs and within models of care has not been clearly defined, potentially explaining the under-inclusion of ENs within integrated models of care [52].

A critical service that was included in most implemented and recommended-only models is care coordination for PLWE. Care coordinators, often ENs, social workers, or designated case managers, link PLWE, families, and stakeholders within and across sectors, facilitating shared care goals across the continuum of care, and are thus central to achieving value-based person-centred care. Psychosocial services (delivered by allied health, specialists, and ENs) were also widely incorporated in models, highlighting strong awareness of the mental comorbidities and psychosocial impacts of epilepsy [43]. CMPs were also included in most implemented and recommended-only models as a way to consolidate shared care goals and improve communication of care needs between PLWE, families, and providers [41].

Several barriers to effective interprofessional collaboration and coordination of care services were reported in the literature. HCPs often lack a comprehensive understanding of their respective roles and responsibilities in epilepsy management, worsened by a lack of structure and processes to support cross-sector communication and learning [34,50]. In addition, a perception of clinical autonomy and territorialism within professions can prevent HCPs from exchanging knowledge, which can limit optimal care for PLWE [6,50]. A key enabler of cross-boundary collaboration in epilepsy management is the development and use of CPGs and care pathways, which can provide direction to HCPs and assist with standardising and effectively coordinating care [38]. In the current review, a greater number of recommended-only models incorporated CPGs and care pathways, compared to implemented models. Barriers associated with implementing CPGs and care pathways include a lack of education and training for HCPs on care protocols [39], limited motivation for change [38], resource and time constraints [47], and coordination issues when involving carers and nonclinical staff (e.g., school personnel) [39]. Greater efforts to collaboratively develop and effectively implement CPGs is necessary to ensure high-quality, safe, and timely epilepsy care [38].

Information and communications technology can also improve information exchange between HCPs and encourage cross-sector collaboration [42,48]. Shared EMRs, featured in seven models identified in this review, enabled joint access to medical records to facilitate shared, coordinated, and compliant care, and improved communication between providers [34]. The implementation of shared EMRs can be challenged by the incompatibility of health information systems across services, requiring governance support to maximise their value in shared care for PLWE [42,48]. Telehealth services such as telephone advice lines can also support collaboration, and could be particularly useful for information exchange between general practitioners and specialist epilepsy services [34]. However, incorporating telehealth into models of care requires significant resources and can increase the demands on HCPs [24,48], potentially explaining the lack of uptake in telehealth innovations. Relatedly, workplace productivity demands can inhibit person-centred and compassionate care delivery, necessitating a cultural shift in organisational operations and funding for staff to build cultures of capacity [48].

Transforming approaches to workforce development is also key to advancing integrated care. Education for HCPs was included in most recommended-only models of care identified in the current review but in less than half of the implemented models. For complex and chronic conditions like epilepsy, education and training in primary and community care settings should be bolstered to support condition management, prevent ill health, and focus on quality of life and wellbeing [38]. The challenges of cross-disciplinary communication and learning, and of role ambiguity amongst HCPs in integrated epilepsy care points to the need for interdisciplinary education and training in medicine, nursing, allied health professions and social care [34,51]. In addition to investing in community-based training and recruitment (such as for general practitioners with a special interest in epilepsy and ENs), governments and professional bodies should prioritise training in integrated, team-based care, that focuses on collective goals [14,29].

A ‘whole system’ approach is needed to improve integrated care, requiring policy changes that commit necessary resources and governance structures and funding models that support collaboration across organisational and sectoral boundaries [39,48]. The safe interoperability of technology within and across organisations should be a central priority of this approach, to improve equitable access to health information, reduce duplication, and improve efficiency [48,53]. Removing the financial and regulatory barriers that perpetuate fragmented care practices requires dedicated and consistent collaboration among leaders at all health system levels [54].

### Strengths and Limitations

This scoping review is the first to explore the breadth and type of integrated care components that have been implemented and recommended but not yet implemented in epilepsy management. The 15 evidence-informed components identified serve as a blueprint for which to assess the comprehensiveness of models of integrated care that aim to deliver high-quality epilepsy management.

There are several limitations worth noting. First, although our search strategies were designed to comprehensively capture integrated care efforts, it is possible that the interventions reviewed were mostly those shown to be successful, omitting those that were less effective but no less useful in informing approaches to integrating epilepsy care. Furthermore, incorporating key search terms for challenges and enablers may have uncovered greater research on the factors associated with integrated care for under-represented groups, such as Indigenous and culturally and linguistically diverse communities. Relatedly, including articles published only in the English language may have limited information about integrated care efforts in non-English speaking populations.

Second, we aimed to capture the range and type of integrated care components and models that have been recommended-only or implemented, but it is beyond our scope to draw conclusions about the components that are of greatest priority. Examining implementation is important to identifying structural and process barriers and enablers, but a comprehensive systematic review will be required to assess the effectiveness of integrated care interventions on health outcomes. We recommend that future reviews also examine grey literature, which can provide useful insights on broad and emerging topics [55].

Third, we focused on integrated care components and models within their specific context, and there was limited exploration of the factors enabling or impeding implementation across different healthcare systems. A deeper exploration of governance structures and funding models and incentives, as well as the relationships between implementation determinants across contexts, is needed to inform the translatability of components and models, and ultimately, their capacity to scale.

### Conclusion

This scoping review provides a comprehensive international overview of literature examining integrated care in epilepsy management. There is widespread ambition to develop integrated person-centred epilepsy care, but implementation is challenging, exacerbated by persistent sectoral siloes, inefficient information exchange between providers and sectors, and unclear care pathways. Transforming approaches to workforce development and the enforcement of new policies to more effectively regulate care environments is required if the benefits of integrated epilepsy care are to be fully realised.

## Additional Files

The additional files for this article can be found as follows:

10.5334/ijic.7659.s1 Appendix 1. Search string examples.

10.5334/ijic.7659.s2 Appendix 2. Summary of articles included in scoping review.

## List of Abbreviations

CMP; care management plan

CPG; clinical practice guideline

EMR; electronic medical record

EN; epilepsy nurse

HCP; healthcare professional

MDT; multidisciplinary team

PLWE; people living with epilepsy

PRISMA-ScR; Preferred Reporting Items for Systematic Reviews and Meta-Analyses Extension for Scoping Reviews

SDM; shared decision-making
