# Supplementary material for: Integrated Care in Epilepsy Management: A Scoping Review of the Models and Components of Health and Social Care Delivery
Source: Int J Integr Care. 2024 Mar 8;24(1):18. doi: 10.5334/ijic.7659 (PMC10921962; doi:10.5334/ijic.7659)
Supplement: Appendix 1. — Search string examples. [file ijic-24-1-7659-s1.pdf]

Appendix 1. Search string examples

Search string from PubMed

(epilepsy[MeSH Terms]) AND ("shared care"[Title/Abstract] OR "Integrated care"[Title/Abstract] OR "Coordinated care"[Title/Abstract] OR "Managed care"[Title/Abstract] OR "Person-Centered"[Title/Abstract] OR "Patient-centered"[Title/Abstract] OR "Person-Centred"[Title/Abstract] OR "Patient-centred"[Title/Abstract])

Search String from Web of Science

Epilepsy [topic] AND (“Shar* care” OR “Integrat* care” OR “Coordinat* care” OR “Manag* care” OR “Person-Cent* care” OR “Patient-Cent* care”)[topic]
