# Supplementary material for: Integrated Care in Epilepsy Management: A Scoping Review of the Models and Components of Health and Social Care Delivery
Source: Int J Integr Care. 2024 Mar 8;24(1):18. doi: 10.5334/ijic.7659 (PMC10921962; doi:10.5334/ijic.7659)
Supplement: Appendix 2. — Summary of articles included in scoping review. [file ijic-24-1-7659-s2.pdf]

Appendix 2. Summary of articles included in scoping review

**Author**	**Country**	**Study design/ Article type**	**Model or component only**	**Objective or aim**
Annear (2019) [36]	UK	Synthesis of expert recommendations	Recommended model	To synthesise existing research on models of care for people with TSC and recommend a new comprehensive model of care.
Auvin (2019) [35]	France	Delphi	Recommended components	To identify key steps in developing an MDT in TSC, and the key functions of the MDT.
Bali (2016) [37]	UK	Literature review	Recommended model	To synthesise existing research on improving epilepsy care for children and young people and recommend a new multisectoral model of care.
Bellon (2014) [16]	Australia	Descriptive	Implemented components	To explore the extent to which PLWE and their family members are, and want to be, involved in the development of their epilepsy management plan.
Berg (2013) [25]	USA	Descriptive	Recommended components	To identify gaps in knowledge and practice in paediatric epilepsy care, and priority areas to improve care processes and outcomes for PLWE.
Buelow (2018) [17]	USA	Literature review, descriptive	Implemented components	To develop and evaluate a tool to facilitate communication between nurses and PLWE using a model of PCC.
Byrne (2019) [48]	Ireland	Descriptive	Implemented model	To explore the readiness of healthcare providers in the Irish epilepsy care system to adopt and benefit from an integrated PCC model.
Cross (2013) [18]	UK	Literature review	Implemented components	To synthesise existing research on managing seizures in children and explore strategies for seizure management in school settings.
Doran (2021) [41]	Ireland	Retrospective cohort	Implemented model	To design a new care pathway for homeless PLWE, improve their access to specialist care, and strengthen links between community and tertiary teams.
Fitzsimmons (2012) [38]	Ireland	Literature review	Recommended model	To synthesise existing research on evidence-based models of care for PLWE and apply the CDM model in evaluating the models of care.
Granata (2011) [26]	Italy	Case series	Recommended components	To report on the potential role of PC comprehensive caring

				throughout the life course and continuum of epilepsy care.
Hafeez (2017) [19]	USA	Descriptive	Implemented components	To explore the content of a care coordination intervention for children with epilepsy at an accountable care organization.
Higgins (2019) [42]	Ireland	Descriptive	Implemented model	To explore how epilepsy specialist nurses enact their clinical role in epilepsy management as members of multidisciplinary teams.
Hutchinson (2020) [6]	Australia	Descriptive	Recommended components	To explore clinical decision-making and shared care practices in managing refractory epilepsy, and the impacts of decisions on care pathways.
Jackson (2022) [39]	USA	Literature review, descriptive	Recommended model	To describe the acute seizure care pathway for children with epilepsy, identify gaps in care, and propose interventions to bridge these gaps.
Kirkpatrick (2022) [27]	USA	Descriptive	Recommended components	To explore the perspectives and experiences of women with epilepsy around PC sexual and reproductive health care provision.
Kluger (2021) [28]	USA	Literature review	Recommended components	To explore the key domains of palliative care relevant to epilepsy that could be adopted to improve models of care for PLWE.
Le Pichon (2022) [20]	USA	Prospective cohort	Implemented components	To explore telehealth initiatives aimed to improve consumer access to and knowledge about epilepsy services.
Lewis (2013) [40]	UK	Case study	Recommended model	To explore the communication and information needs of young people and their caregivers during transition from child to adult epilepsy services.
Lindhart (2021) [21]	Denmark	Descriptive	Implemented components	To examine the impact of a personalised discharge letter in facilitating smooth transition from hospital to home.
Minshall (2021) [29]	UK	Retrospective cohort	Recommended components	To assess the standard of clinical care of PLWE in primary care since the removal of epilepsy from the Quality and Outcomes Framework.
Ogundele (2022) [30]	UK	Narrative review	Recommended components	To synthesise existing research on NDEBIDs and provide recommendations for more integrated approaches to care.

Patel (2017) [43]	USA	Retrospective cohort	Implemented model	To implement and evaluate interventions designed to decrease seizure-related ED presentations.
Power (2020) [49]	Ireland	Descriptive	Implemented model	To understand the care experiences of PLWE and barriers and facilitators to implementing integrated PCC.
Pugh (2020) [44]	Australia	Prospective cohort, retrospective cohort	Implemented model	To evaluate a community-based nursing service supporting people with neurological conditions in their discharge from hospital.
Samanta (2021) [31]	USA	Literature review	Recommended components	To explore the range of interventions available to address the underutilisation of epilepsy surgery.
Satherley (2021) [45]	UK	Descriptive	Implemented model	To understand family perspectives about a new integrated epilepsy care service for children and young people.
Saxena (2022) [32]	USA	Literature review	Recommended components	To synthesise evidence on the biopsychosocial model to facilitate PCC for neurological conditions across outpatient and inpatient settings.
Scavasine (2022) [24]	Brazil	Retrospective cohort	Implemented components	To evaluate the efficacy of a store-and-forward teleneurology system in general practice.
Tetuan (2019) [22]	USA	Retrospective cohort	Implemented components	To evaluate a novel interprofessional telehealth program designed to improve care access for rural-dwelling PLWE.
Tschamper (2019) [23]	Norway	Descriptive	Implemented components	To evaluate videoconferencing as a collaborative information exchange platform for MDTs and families.
Tumiene (2022) [33]	Lithuania	Narrative review	Recommended components	To synthesise existing research on PC integrated care approaches for people with inherited metabolic diseases.
Varley (2010) [34]	Ireland	Descriptive	Recommended components	To explore the perspectives and experiences of HCPs in primary and specialist epilepsy care services.
Varley (2020) [50]	Ireland	Descriptive	Implemented model	To explore challenges and opportunities for community-hospital partnerships in epilepsy care.
Williams (2017) [47]	Ireland	Descriptive	Implemented model	To identify the barriers to implementing an ED integrated care pathway for seizure

				management.
Williams (2018) [46]	Ireland	Retrospective cohort	Implemented model	To evaluate the utilisation and implementation of an ED integrated care pathway for seizure management.

UK, United Kingdom; USA, United States of America; TSC, tuberous sclerosis complex; MDT, multidisciplinary team; PLWE, people living with epilepsy; PCC, person-centred care; CDM, chronic disease management; ED, emergency department; NDEBIDs, neurodevelopmental, emotional, behavioural, and intellectual disorders; HCPs, healthcare professionals.
